# Growth and Mortality of Zoo‐Reared Ozark Hellbenders, *Cryptobranchus alleganiensis bishopi* (Grobman 1943)

**DOI:** 10.1002/zoo.21870

**Published:** 2024-11-26

**Authors:** D. Cristina Macklem, Lauren Augustine, Mark D. Wanner, Jeffery A. Ettling, Trisha Crabill, Amanda S. Pedigo, Chawna Schuette, Patty L. Ihrig‐Bueckendorf, Aja J. Martin, Katie R. Noble, Justin M. Elden, Jeffrey T. Briggler

**Affiliations:** ^1^ Saint Louis Zoo St. Louis Missouri USA; ^2^ Philadelphia Zoo Philadelphia Pennsylvania USA; ^3^ Smithsonian National Zoological Park Washington District of Columbia USA; ^4^ Brookfield Zoo Brookfield Illinois USA; ^5^ Jacksonville Zoo and Gardens Jacksonville Florida USA; ^6^ U.S. Fish and Wildlife Service Columbia Missouri USA; ^7^ No Leash Needed O'Fallon Illinois USA; ^8^ Missouri Department of Conservation Jefferson City Missouri USA

**Keywords:** common garden, conservation breeding, head‐starting, salamander, species recovery plan

## Abstract

Ozark hellbender (*Cryptobranchus alleganiensis bishopi*, Grobman 1943) populations in Missouri and Arkansas have been federally listed as endangered since 2011. As part of the comprehensive recovery plan for the subspecies, the Saint Louis Zoo WildCare Institute's Ron and Karen Goellner Center for Hellbender Conservation, in collaboration with the Missouri Department of Conservation, Arkansas Game and Fish Commission, and the U.S. Fish and Wildlife Service, established a conservation breeding and head‐starting program to augment and create self‐sustaining wild populations. We examined how the river of origin and egg origin (i.e., Zoo‐bred or wild‐bred) influenced various growth and mortality responses of Zoo‐reared Ozark hellbenders. River of origin significantly predicted most larval and long‐term Zoo‐reared Ozark hellbender growth responses, with our results concurring with observed differences in wild populations and known genetic relationships between Ozark hellbender populations. Mortality of Zoo‐reared Ozark hellbenders was often predicted by river of origin and egg origin with Zoo‐bred hellbenders having significantly higher proportional mortality responses relative to wild‐bred hellbenders. Further exploration of this egg origin relationship revealed differences between the Zoo breeding group generations with higher proportional mortality rates for hellbenders from the less mature second‐generation breeding group relative to hellbenders from the first‐generation breeding group and wild‐bred hellbenders. Ultimately, our results provide baseline data on Zoo‐bred and wild‐bred Ozark hellbenders in the program, help identify differences in growth and mortality responses between Ozark hellbender populations, and contribute to existing evidence supporting distinct populations of Ozark hellbenders in Missouri to aid in targeted conservation strategies.

## Summary

Our results indicate that larval and long‐term growth of Ozark hellbenders raised at the Saint Louis Zoo differ by river of origin in accordance with observations from wild populations and genetic relatedness.

## Graphical abstract

The long‐term growth of Ozark hellbenders raised at the Saint Louis Zoo differ by river of origin (EPR, CR, and NFR, as Eleven Point River, Current River, and North Fork of the White River, respectively) in accordance with observations from wild populations and genetic relatedness.

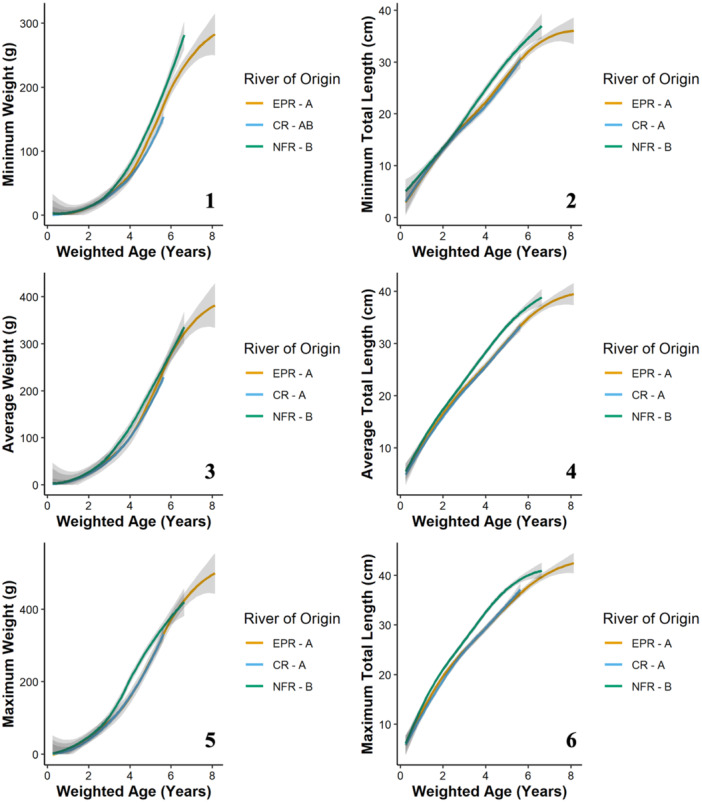

## 1 Introduction

Ozark hellbender (*Cryptobranchus alleganiensis bishopi*, Grobman 1943) populations in Missouri and Arkansas have been federally listed as endangered since 2011 with Missouri populations being listed as state endangered since 2003 (U.S. Fish and Wildlife Service 2011). Habitat degradation and loss, illegal collection, disease, reduced water quality, and predation by native and nonnative fish are likely the primary drivers of population declines (Briggler et al. 2010; U.S. Fish and Wildlife Service 2020, 2021). With declining populations, a comprehensive recovery plan was developed and finalized in 2010 and later updated and replaced by the federal recovery plan in 2021 (Briggler et al. 2010; U.S. Fish and Wildlife Service 2021). These plans included mitigating the impacts of habitat degradation and disease; enhancing protection, research, and education efforts; and establishing a conservation breeding and head‐starting program with the goal of augmenting extant populations (Briggler et al. 2010, 2012; U.S. Fish and Wildlife Service 2021).

When Ozark hellbender populations declines were initially documented, assessing the genetic diversity and relatedness of Ozark hellbender populations became a priority to determine the genetic viability of remaining populations and create targeted conservation goals for distinct genetic lineages (Briggler et al. 2010). The earliest genetic analyses showed low levels of within‐population variation and distinct genetic signatures between populations (Templeton et al. 1990; Routman 1993; Kucuktas et al. 2001). Later studies quantified genetic (mitochondrial and genomic) relatedness between Ozark hellbender populations. These studies showed that Ozark hellbenders from the Current River and Eleven Point River were genetically distinct from other populations of Ozark hellbenders (Sabatino and Routman 2009; Crowhurst et al. 2011; Tonione, Johnson, and Routman 2011; Hime 2017). Thus, while being classified as the same subspecies, maintaining these unique genetic lineages became an important component of conservation efforts for Ozark hellbenders (Briggler et al. 2012).

With the identification of conservation breeding and head‐starting efforts as a primary mechanism for augmenting wild Ozark hellbender populations (Briggler et al. 2010, 2012; U.S. Fish and Wildlife Service 2021), the Saint Louis Zoo (Zoo) and its partners, the Missouri Department of Conservation (MDC), U.S. Fish and Wildlife Service, and Arkansas Game and Fish Commission, established the Ron and Karen Goellner Center for Hellbender Conservation (Center) at the Zoo in 2003. With time, the Center created a comprehensive head‐starting and conservation breeding program that includes three artificial streams for breeding, four rooms in the basement of the Charles H. Hoessle Herpetarium dedicated to rearing hellbenders, integrated water quality and life support systems, and protocols on husbandry, veterinary care, and propagation of hellbenders (Junge 2011; Briggler et al. 2012; Ettling et al. 2013; Pedigo et al. 2021). The goal of the Center is to breed, rear, and augment wild Ozark hellbender populations until there are self‐sustaining Ozark hellbender populations in native rivers (Briggler et al. 2010, 2012; U.S. Fish and Wildlife Service 2021). In 2011, the Zoo became the first to successfully breed Ozark hellbenders (Ettling et al. 2013), and, in 2018, the Saint Louis Zoo became the only organization to successfully reproduce second‐generation Ozark hellbenders (Saint Louis Zoo WildCare Institute 2018). The MDC first started augmenting wild Ozark hellbender populations with hellbenders reared at the Zoo in 2008, and by 2020, a total of 7975 Ozark hellbenders had been released into four native rivers. The longevity of the propagation program and its success maintaining unique genetic lineages of Ozark hellbenders at the Zoo provides a unique opportunity to learn more about the biology of Ozark hellbenders as well as conservation breeding and head‐starting efforts for the subspecies.

Our objective was to evaluate potential biological differences in larval growth, long‐term growth, and mortality of Ozark hellbenders reared at the Zoo (i.e., Zoo‐reared). We compared these responses to the hellbender river of origin (i.e., unique hellbender populations) as well as egg origin (i.e., eggs from hellbenders that were bred at the Zoo vs. eggs collected from the wild, hereafter Zoo‐bred and wild‐bred, respectively). The goal of this research was to provide baseline growth and survival data on Zoo‐bred and wild‐bred Ozark hellbenders in the program, help identify potential biological differences between Ozark hellbender populations, and contribute to existing evidence supporting distinct populations of Ozark hellbenders to aid in targeted conservation strategies. We hypothesized that river of origin would be the primary predictor of the larval growth, long‐term growth, and mortality responses due to genetic differences between each of the rivers of origin. We did not anticipate that egg origin would be a significant predictor of any of the growth or mortality responses except egg mortality due to different developmental conditions between Zoo‐and wild‐bred eggs.

## 2 Methods

### 2.1 Study Species

The Ozark hellbender is a fully aquatic salamander that has historically occupied several rivers in the Ozark Plateau region of Missouri and Arkansas including the Spring River, North Fork of the White River, Bryant Creek, Jack's Fork River, Eleven Point River, and Current River (U.S. Fish and Wildlife Service 2020). The hellbenders from the study originate from the North Fork of the White River, Current River, and Eleven Point River, all three of which are spring‐fed rivers that have large portions of riparian habitat managed and protected by either the Mark Twain National Forest and/or Ozark National Scenic Riverways (U.S. Fish and Wildlife Service 2020). Hellbenders consume a diet of crayfish, fish, and macroinvertebrates, and reside under large cover objects or within rock crevices in the river (Briggler and Johnson 2021). With the exception of Ozark hellbenders from the Spring River, which have been documented breeding as late as January (Peterson, Ingersol, and Wilkinson 1989), Ozark hellbenders typically breed from late September to late October (Peterson et al. 1983; Briggler and Johnson 2021). During the breeding season, male hellbenders excavate nests under rock cover or within bedrock (Smith 1907; Nickerson and Mays 1973; Settle, Briggler, and Mathis 2018). Females will enter nest chambers to oviposit eggs, which the males will fertilize externally (Smith 1907; Nickerson and Mays 1973; Settle, Briggler, and Mathis 2018). The male continues to provide parental care throughout egg development by oxygenating the eggs, protecting them from predation, and tending to the nest (Smith 1907; Nickerson and Mays 1973; Settle, Briggler, and Mathis 2018). Hellbender eggs typically complete development in approximately 45 to 75 days (Smith 1907; Green and Pauley 1987; Petranka 1998), and offspring reach sexual maturity between 5 and 8 years old (Bishop 1941; Dundee and Dundee 1965).

### 2.2 Husbandry

All Ozark hellbenders were housed and cared for in the Center in the Charles H. Hoessle Herpetarium at the Zoo. Eggs oviposited at the Zoo are not removed from nest boxes until approximately 12–14 days after fertilization. Eggs are collected from nests, either from the wild or from the Zoo, and are transferred to an egg tray system equipped with a chiller, UV sterilization, and filtration system that help to regulate water quality. In addition to the filter pumps, which gently move the eggs like flowing water, caretakers also manually “rock” the eggs 3–4 times a day to imitate tail fanning behavior typically provided by brooding males (Okada, Fukuda, and Takahashi 2015; Settle, Briggler, and Mathis 2018). After hatching and as they grow, hellbenders are transferred to increasingly large enclosures including various sized Kritter Keepers and 20‐ and 40‐gallon aquarium tanks, which may be outfitted with gravel substrate, Siporax biofilter medium, and/or hides depending on their age and size. Water quality parameters known to influence physiology and health are closely monitored and maintained at all stages and reflect conditions found in the wild as much as possible. The water turnover rate for the system is approximately 5–10 times per hour, which helps to maintain water quality and mimics water flow in the system. All nonbreeding hellbenders are kept at a constant temperature of 15.5°C–17°C (60°F–62°F) year‐round and experience light/dark cycles daily. The diet of hellbenders at the Zoo is designed to be as nutritionally comparable to the natural diet as possible and is adjusted as the animals age (Ettling et al. 2013). See Pedigo et al. 2021, for more detailed husbandry and care protocols.

### 2.3 Analysis

We used an information theoretic approach to assess how river of origin and egg origin influence the larval growth, long‐term growth, and mortality of Zoo‐reared Ozark hellbenders (Burnham and Anderson 2002). We acknowledge that the care provided by the Zoo is not intended to and cannot replicate a fully randomized experimental design and that husbandry protocols may vary in accordance with available resources at the Zoo and current conservation priorities. However, husbandry protocols sought to provide consistent and standardized care for nonbreeding Ozark hellbenders by tightly maintaining environmental and water quality parameters as well as providing age‐appropriate diets, enclosure designs, and tank densities (Pedigo et al. 2021). We attempted to account for variation in care in our modeling approach by including clutch year as a random variable when appropriate. Hellbenders from several clutches have been used for targeted recovery experimentation throughout the years. We did not include data from any hellbenders involved in experiments; however, if only partial clutches were used for experiments, we retained data for the nonexperimental hellbenders in those clutches. This occurred for four clutches. We also censored long‐term growth responses for one Ozark hellbender clutch following their involvement in an experiment as juveniles. The conditions at the Zoo equalize much of the typical environmental variation that could influence the biological responses of hellbenders. Thus, we used this modified common garden design to examine potential differences in growth and mortality responses (de Villemereuil et al. 2016).

We defined a clutch, our unit for analyses, as all eggs (jelly coat and ovum) collected from a brooding male in a given reproductive year. We are unable to know the parentage of Zoo or wild eggs without genetic testing; however, the brooding male has always known. We assumed that eggs belonged to the brooding male because wild Cryptobranchid males territorially guard and tend to nests (Smith 1907; Settle, Briggler, and Mathis 2018; Unger et al. 2020, 2021). Moreover, when a male Cryptobranchid is known to have fertilized a clutch, they exhibit parental care behaviors and site fidelity to the nest (Ettling et al. 2013; Luo et al. 2018). We quantified the average age of hellbenders from a clutch at any given measurement period (i.e., any time morphological measurements were collected for a clutch) based on the measurement date and a weighted hatch date for each clutch. We calculated the weighted hatch date, henceforth hatch date, by weighing each individual hatch date by the number of eggs that hatch. We opted to use this value because not all eggs in a clutch hatch on the same day, and the mean hatch date isn't necessarily reflective of peak hatching dates. In some cases, zookeepers may manually hatch eggs to avoid hatchling death, and we used the manual hatch date for these individuals because we assumed that this practice was not performed in an intentionally biased manner. We used a similar method to calculate a weighted age, henceforth age, at the time of measurement if individuals from a clutch were unable to all be measured on the same exact day in a given measurement period.

Larval growth response variables for Zoo‐reared Ozark hellbenders included the minimum, maximum, and average weight and total length (i.e., snout to tail tip) of individuals from each clutch. Larval growth response variables were calculated as an average, one‐time measurement of all individuals from a clutch. At the Zoo, hellbenders were typically measured for the first time just over a year after hatching. We selected this age period because, in addition to being the first measurement for individuals in most clutches, it was also the last measurement before any individuals were released into the wild. Thus, this measurement period represents the only time where all surviving individuals from a clutch are measured. We hypothesized that North Fork of the White River individuals would be larger than either Current River or Eleven Point River individuals due to observations from wild populations (Peterson et al. 1983, 1988; Ziehmer and Johnson 1992; Wheeler et al. 2003) and genetic relatedness between the populations (Sabatino and Routman 2009; Crowhurst et al. 2011; Tonione, Johnson, and Routman 2011; Hime 2017). We also hypothesized that weighted age at the time of measurement would be significant for all larval growth responses but that egg origin would not be a significant predictor of any larval growth responses.

Similarly to larval growth, we examined the minimum, maximum, and average weight and total length as long‐term growth (i.e., growth from the first measurement period to the final measurement period before release into the wild or transfer to the Zoo's breeding groups) responses for Zoo‐reared Ozark hellbenders with the goal of being able to compare long‐term growth trends in Zoo‐reared Ozark hellbenders. We calculated each response variable as an average of all individuals from a clutch at each measurement period. Typically, clutches are measured once a year. We acknowledge that individuals were released into the wild throughout these long‐term data, which could confound the results of the long‐term growth analyses. Additionally, release priorities and selection processes differed from year to year based on the needs of the wild populations as well as the space and resources of the Zoo, which means that the individuals selected for release were sometimes a nonrandom sample of individuals from a clutch. However, as a program with the goal of breeding and head‐starting an endangered subspecies, these are the only long‐term growth data available, and we made efforts to minimize confounding effects where possible. For example, most measurement dates preceded releases, which meant that all individuals, including those slated for release, were measured at each time period. The remaining individuals were typically not measured again until the following year, further reducing the potential for acute changes to the response variable values due to releases. Moreover, all growth measurements represent an average of all remaining individuals from a clutch at the time of measurement. These averages help to buffer potential high‐magnitude changes. Lastly, due to increased uncertainty with fewer and fewer individuals, we censored measurements after fewer than five individuals from a clutch remained. As with our larval growth responses, we hypothesized that North Fork of the White River individuals would continue to be larger than either Current River or Eleven Point River individuals for all long‐term growth responses, weighted age at the time of measurement would be significant predictor for all long‐term growth responses, and egg origin would not be a significant predictor of long‐term growth responses.

We quantified the proportional mortality of Zoo‐reared hellbenders at three life stages: egg, hatchling, and larva. We calculated proportional mortality metrics to allow for comparison between differently sized clutches. We added any egg death or egg removal weighted by the original clutch size to calculate the proportional egg mortality. Egg removals occurred because of infertility (i.e., a failure to develop), infection, or congenital deformities that were incompatible with life. We include infertile eggs in our egg mortality calculation as a way to account for the total reproductive cost to the breeding individuals and the failure of that reproductive investment. There is a degree of uncertainty for the proportional egg mortality response because, for wild‐bred eggs, a portion of egg development occurred in the wild, where any mortalities that occurred before arrival at the Zoo were unaccounted for in the data presented here. Similarly, Zoo‐oviposited eggs typically aren't removed from brooding males until at least 12–14 days after fertilization (Pedigo et al. 2021), during which time unaccounted for cannibalization and predation could occur. As a result, egg mortality estimates are likely underestimations. We added any deaths post‐hatching up to 2 months (60 days) of age weighted by the original number of hatchlings to calculate the proportional hatchling mortality response. We identified this as a unique and important life stage to include because hatchlings are entirely reliant on their yolk sacs for nutrition, relatively sedentary, and experiencing morphological changes as they develop (Smith 1907, 1912). We added any deaths from 60 days old to 1‐year post‐hatch date weighted by the original larval count to calculate the proportional larval mortality. Lastly, we calculated the total proportional mortality by adding mortalities across all life stages weighted by the original clutch size. We hypothesized that there may be differences in proportional mortality due to potential differences in the genetic viability of each of the rivers of origin. We hypothesized that egg origin would not influence proportional mortality except at the egg life stage. We expected lower proportional egg mortality for wild‐bred eggs due to a longer duration of time under the care of a brooding male, coming to the Zoo at a more advanced developmental stage, and being unable to account for mortalities from early egg development.

We created regression models to examine larval growth, long‐term growth, and proportional mortality responses for Zoo‐reared Ozark hellbenders. For all models, we assessed model assumptions, and, for response variables that did not fit a Gaussian distribution, we fit the models to a Gamma, lognormal, or Poisson distribution. We created one trivariate regression model for each of our Zoo‐reared Ozark hellbender larval and long‐term growth response variables. Each model included age at the time of measurement as a predictor variable because not all clutches were measured at the same age. We also included two predictive factor variables, river of origin and egg origin, and included reproductive year as a random effect. Total lengths for Ozark hellbenders were modeled using Gaussian distributions while weights were modeled using either log normal or Gamma distributions. To examine proportional mortality responses, we created bivariate regression models for Ozark hellbenders, which included river of origin and egg origin as well as the reproductive year as a random effect. We multiplied all proportional mortalities by 100 and modeled these regressions as Poisson distributions.

We performed type II Wald chi‐squared tests to assess the significance of predictor variables to our response variables, and we performed Tukey post hoc tests to assess differences within predictive factor variables. Data were analyzed using program R (version 3.6.3) through the RStudio (version 1.2.5033) interface.

## 3 Results

We included 46 Ozark hellbender clutches that were laid between 2012 and 2020 in our larval growth and proportional mortality analyses. Eleven Point River and North Fork of the White River were the rivers of origin for 14 clutches each. Current River was the river of origin for 18 clutches. A total of 20 clutches originated at the Zoo while 26 clutches originated from eggs collected in the wild.

Due to the temporal scale of the long‐term growth analyses, we were only able to include 39 Ozark hellbender clutches that were laid between 2012 and 2019. Eleven Point River was the river of origin for 12 clutches (1807 individuals). Current River was the river of origin for 15 clutches (1563 individuals), and North Fork of the White River was the river of origin for 12 clutches (3161 individuals). A total of 16 clutches originated at the Zoo while 23 clutches originated in the wild.

### 3.1 Larval Growth

**Figure 1:**
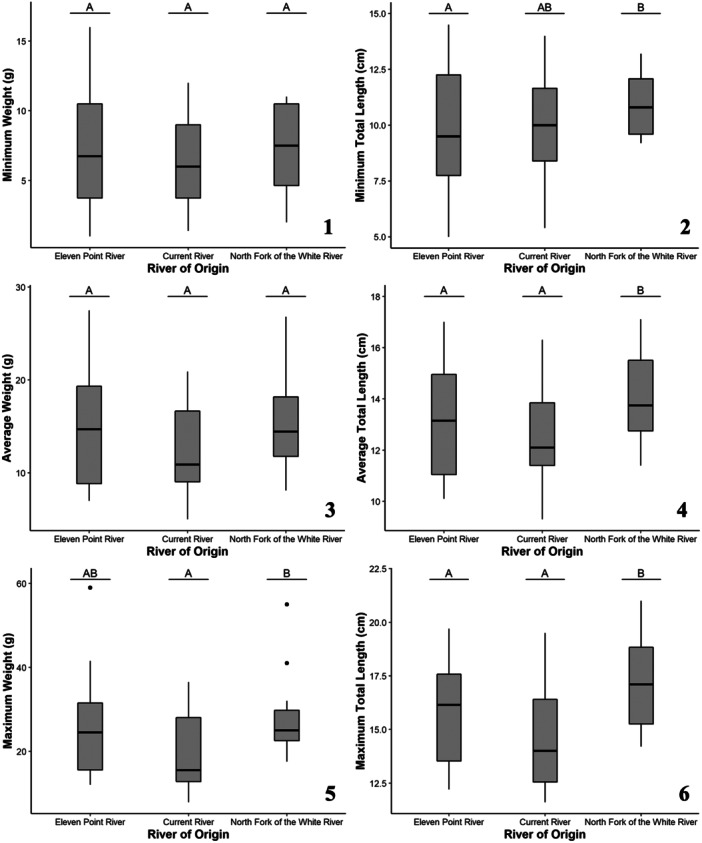
The larval growth responses of all Zoo‐reared (i.e., Zoo‐bred and wild‐bred) Ozark hellbenders including minimum (1), average (3), and maximum (5) weights as well as minimum (2), average (4), and maximum (6) total lengths as a function of the predictive factor, river origin. Lettering over box plots denotes differences between factor levels with unique letters indicating significant differences while multiple letters indicate values that are indistinguishable from either unique letter.

**Figure 2:**
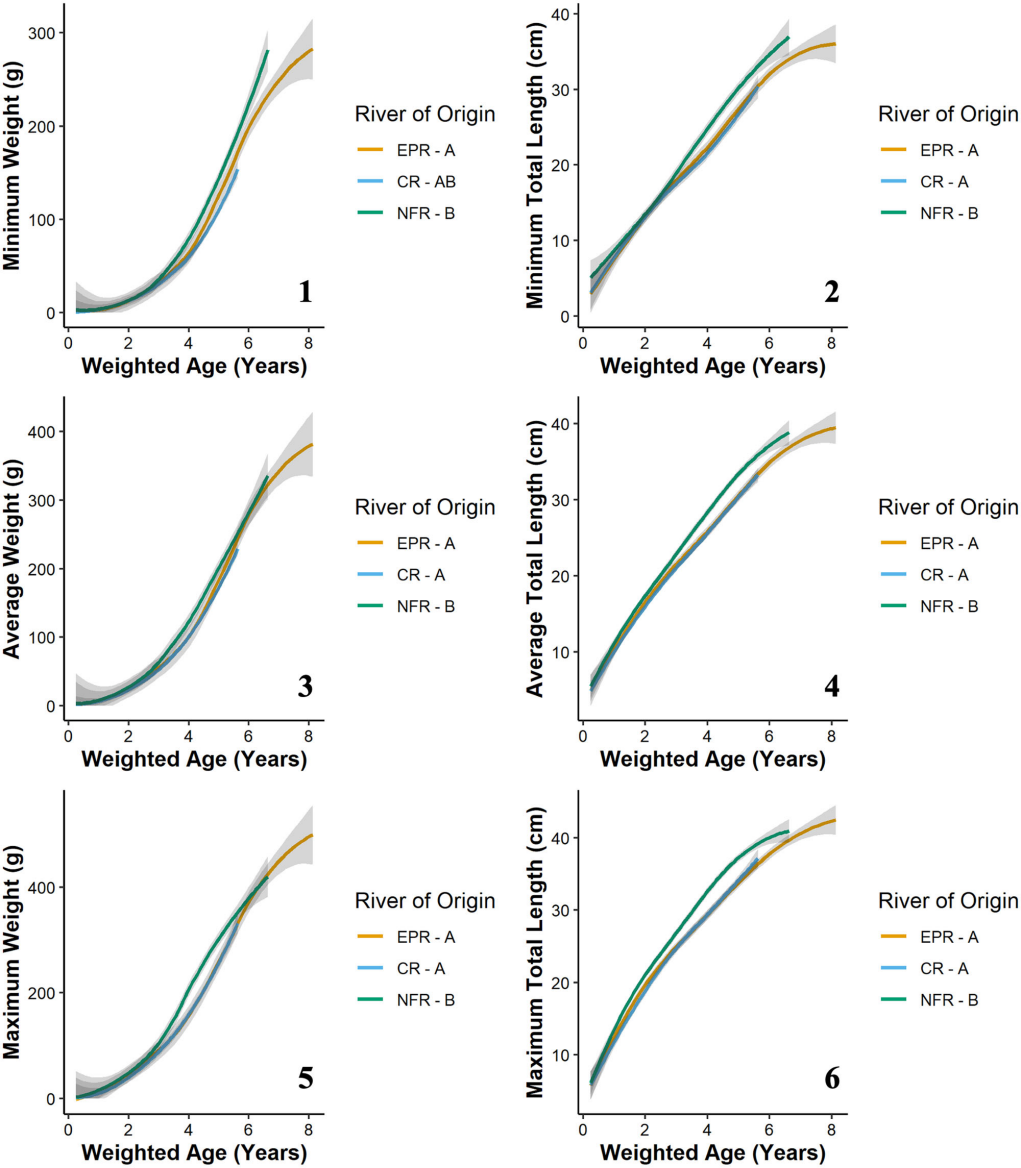
The long‐term growth responses of all Zoo‐reared (i.e., Zoo‐bred and wild‐bred) Ozark hellbenders including minimum (1), average (3), and maximum (5) weights as well as minimum (2), average (4), and maximum (6) total lengths as a function of the predictive factor, river origin. The acronyms EPR, CR, and NFR indicate individuals originating from the Eleven Point River, Current River, and North Fork of the White River, respectively. Lettering next to the river of origin acronyms denotes differences between factor levels with unique letters indicating significant differences while multiple letters indicate values that are indistinguishable from either unique letter.

**Figure 3:**
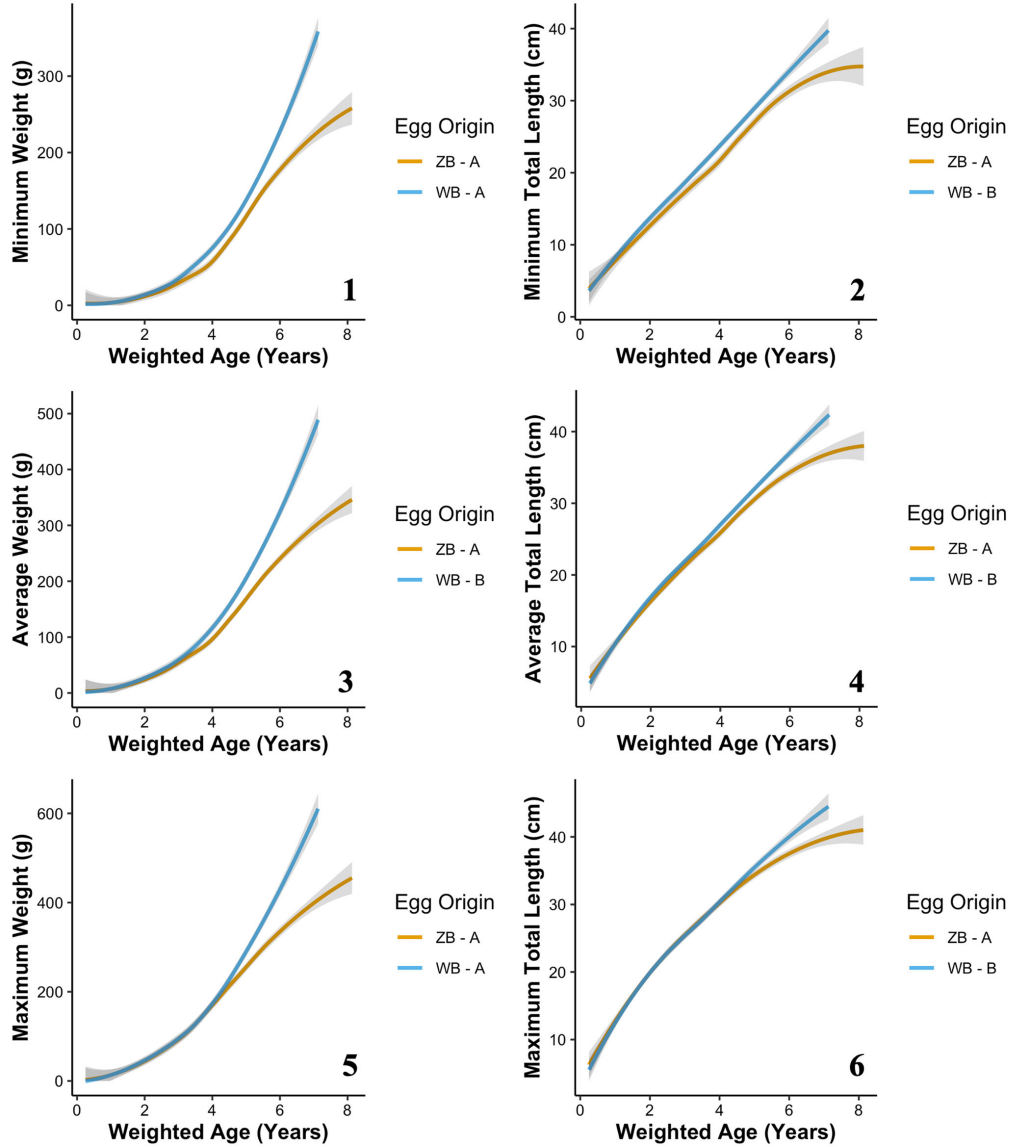
The long‐term growth responses of Zoo‐reared Ozark hellbenders from all rivers of origin including minimum (1), average (3), and maximum (5) weights as well as minimum (2), average (4), and maximum (6) total lengths as a function of the predictive factor, egg origin. The acronyms ZB and WB indicate Zoo‐bred and wild‐bred egg origins, respectively. Lettering next to the egg origin acronyms denotes differences between factor levels with unique letters indicating significant differences.

**Figure 4:**
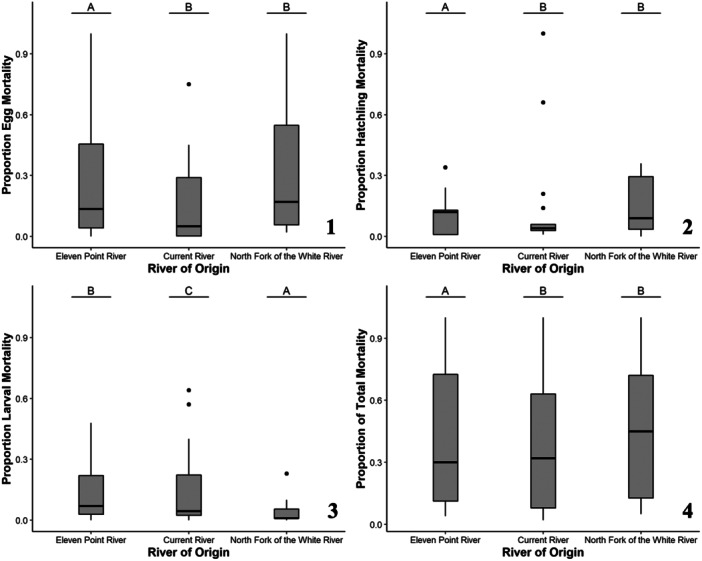
The mortality responses for all Zoo‐reared (i.e., Zoo‐bred and wild‐bred) Ozark hellbenders including proportional egg (1), hatchling (2), larval (3), and total (4) mortality as a function of the significant predictive factor, river origin. Lettering over box plots denotes differences between factor levels with unique letters indicating significant differences.

**Figure 5:**
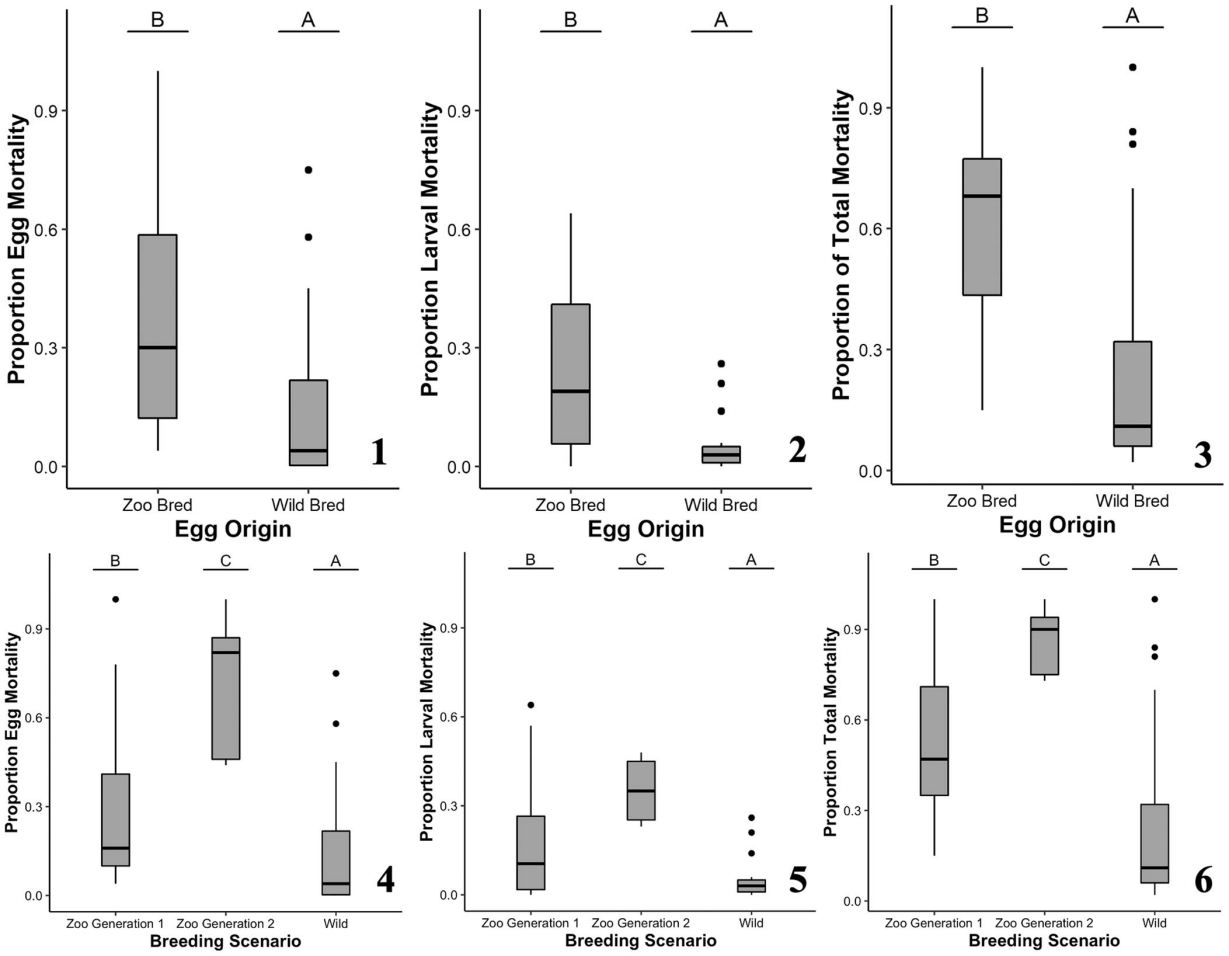
The mortality responses for Zoo‐reared Ozark hellbenders from all rivers of origin including proportional clutch (1), larval (2), and total (3) mortality as a function of the significant predictive factor, egg origin and then further explored as breeding scenario (4, 5, 6). Lettering over box plots denotes differences between factor levels with unique letters indicating significant differences.

Average age for Ozark hellbenders at the time of measurements was 1.4 years old (SD: 0.3, minimum: 1.1 years old, maximum: 2.0 years old). The average of average weights of Ozark hellbenders was 13.9 g (minimum: 1.0 g, maximum: 59.0 g). Minimum weight was significantly predicted by age at the time of measurement (*χ*² = 28.06, *p* < 0.001). Neither river of origin nor egg origin were significant predictors of minimum weight (*χ*² = 0.82, *p* = 0.663; *χ*² = 1.66, *p* = 0.198; Figure 1‐1). Average weight was significantly predicted by age at the time of measurements (*χ*² = 60.13, *p* < 0.001). Neither river of origin nor egg origin were significant predictors of average weight (*χ*² = 4.87, *p* = 0.087 *χ*² = 0.10, *p* = 0.747; Figure 1‐3). Maximum weight was significantly predicted by age at the time of measurements (*χ*² = 53.97, *p* < 0.001) and river of origin (*χ*² = 9.04, *p* = 0.011; Figure 1‐5). Current River individuals had significantly smaller maximum weights relative to North Fork of the White River individuals. Eleven Point River individuals fell in between the maximum weights of Current River and North Fork of the White River individuals. Egg origin was not a significant predictor of maximum weight (*χ*² = 1.71, *p* = 0.191).

The average of average total lengths of Ozark hellbenders was 13.2 cm (minimum: 5.0 cm, maximum: 21.0 cm). Minimum total length was significantly predicted by age at the time of measurements (*χ*² = 64.97, *p* < 0.001) and river of origin (*χ*² = 7.33, *p* = 0.026; Figure 1‐2). Eleven Point River individuals had significantly smaller maximum total lengths relative to North Fork of the White River individuals. Current River individuals had maximum total lengths in between Eleven Point River and North Fork of the White River individuals. Egg origin was not a significant predictor of minimum total length (*χ*² = 2.16, *p* = 0.142). Average total length was significantly predicted by age at the time of measurements (*χ*² = 113.54, *p* < 0.001) and river of origin (*χ*² = 23.81, *p* < .001; Figure 1‐4). Current River and Eleven Point River individuals had significantly smaller average total lengths relative to North Fork of the White River individuals. Egg origin was not a significant predictor of average total length (*χ*² = 0.12, *p* = 0.734). Maximum total length was significantly predicted by age at the time of measurements (*χ*² = 142.88, *p* < 0.001) and river of origin (*χ*² = 26.92, *p* < 0.001; Figure 1‐6). Current River and Eleven Point River individuals had significantly smaller maximum total lengths relative to North Fork of the White River individuals. Egg origin was not a significant predictor of maximum total length (χ² = 0.97, *p* = 0.325).

### 3.2 Long‐Term Growth

The long‐term growth data for Ozark hellbenders included 102 measurement periods from Eleven Point River hellbenders, 96 from Current River hellbenders, and 83 from North Fork of the White River hellbenders. A total of 132 measurement periods were of Zoo‐bred hellbenders, and 149 were of wild‐bred hellbenders. The average age at the time of measurement was 3.2 years old. The youngest individuals measured were 0.3 years old and the oldest individuals measured were 8.1 years old.

The long‐term minimum weight of Zoo‐reared Ozark hellbenders was significantly predicted by age at the time of measurement (*χ*² = 2326.06, *p* < 0.001) and river of origin (*χ* = 7.706, *p* = 0.029; Figure 2‐1). Eleven Point River individuals had significantly smaller minimum weight measurements relative to North Fork of the White River individuals, and Current River individuals had intermediate weights. Egg origin was not significant (*χ*² = 1.27, *p* = 0.259; Figure 3‐1). The long‐term average weight of Zoo‐reared Ozark hellbenders was significantly predicted by age at the time of measurement (*χ*² = 2524.88, *p* < 0.001), river of origin (*χ*² = 12.23, *p* = 0.002; Figure 2‐3), and egg origin (*χ*² = 5.87, *p* = 0.015; Figure 3‐3). Current River and Eleven Point River individuals had significantly smaller average weight measurements relative to North Fork of the White River individuals, and Zoo‐bred hellbenders had significantly smaller average weight measurements relative to wild‐bred hellbenders. The long‐term maximum weight of Zoo‐reared Ozark hellbenders was significantly predicted by age at the time of measurement (*χ*² = 1616.93, *p* < 0.001) and river of origin (*χ*² = 15.07, *p* = 0.001; Figure 2‐5). Current River and Eleven Point River individuals had significantly smaller minimum weight measurements relative to North Fork of the White River individuals. Egg origin was not significant (*χ*² = 15.36, *p* < 0.001; Figure 3‐5).

The long‐term minimum total length of Zoo‐reared Ozark hellbenders was significantly predicted by age at the time of measurement (*χ*² = 5070.95, *p* < 0.001), river of origin (*χ*² = 47.27, *p* < 0.001; Figure 2‐2), and egg origin (*χ*² = 10.52, *p* = 0.001; Figure 3‐2). Current River and Eleven Point River individuals had significantly shorter minimum total length measurements relative to North Fork of the White River individuals, and Zoo‐bred hellbenders had significantly shorter minimum total length measurements relative to wild‐bred hellbenders. The long‐term average total length of Zoo‐reared Ozark hellbenders was significantly predicted by age at the time of measurement (*χ*² = 7608.19, *p* < 0.001), river of origin (*χ*² = 91.87, *p* < 0.001; Figure 2‐4), and egg origin (*χ*² = 15.36, *p* < 0.001; Figure 3‐4). Current River and Eleven Point River individuals had significantly shorter minimum total length measurements relative to North Fork of the White River individuals, and Zoo‐bred hellbenders had significantly shorter minimum total length measurements relative to wild‐bred hellbenders. The long‐term maximum total length of Zoo‐reared Ozark hellbenders was significantly predicted by age at the time of measurement (*χ*² = 4253.16, *p* < 0.001), river of origin (*χ*² = 78.51, *p* < 0.001; Figure 2‐6) and egg origin (*χ*² = 8.04, *p* = 0.018; Figure 3‐6). Current River and Eleven Point River individuals had significantly shorter maximum total length measurements relative to North Fork of the White River individuals, and Zoo‐bred hellbenders had significantly shorter maximum total length measurements relative to wild‐bred hellbenders.

### 3.3 Mortality

Ozark hellbender mortality decreased at every subsequent life stage with average egg mortality at 26%, average hatchling mortality at 13%, and average larval mortality at 12%. The average total proportional mortality across all life stages was 41%.

River of origin was a significant predictor for the proportion of egg mortality (*χ*² = 49.27, *p* < 0.001; Figure 4‐1) with Eleven Point River individuals having a lower proportion of egg mortality relative to Current River and North Fork of the White River individuals. Egg origin was also a significant predictor of the proportion of egg mortality (*χ*² = 175.57, *p* < 0.001; Figure 5‐1) with wild‐bred eggs having a significantly lower proportion of egg mortality relative to Zoo‐bred eggs. Further exploration of the breeding scenarios (i.e., wild bred, first‐generation Zoo breeding group bred, and second‐generation breeding group bred) determined that wild‐bred eggs had the lowest egg mortality, followed by eggs from the first‐generation Zoo breeding group, then eggs from the second‐generation Zoo breeding group (Figure 5‐4). River of origin was a significant predictor for the proportion of hatchling mortality (*χ*² = 13.14, *p* = 0.001; Figure 4‐2) with Eleven Point River individuals having a lower proportion of hatchling mortality relative to Current River and North Fork of the White River individuals. Egg origin was not a significant predictor of the proportion of hatchling mortality (*χ*² = 0.81, *p* = 0.369). River of origin was a significant predictor for the proportion of larval mortality (*χ*² = 67.42, *p* < 0.001; Figure 4‐3) with North Fork of the White River individuals having the lowest proportion of larval mortality, followed by Eleven Point River individuals, then Current River individuals. Egg origin was also a significant predictor of the proportion of larval mortality (*χ*² = 92.43, *p* < 0.001; Figure 5‐2) with wild‐bred individuals having significantly lower larval mortality than Zoo‐bred individuals. Further exploration of the breeding scenarios determined that wild‐bred hellbenders had the lowest larval mortality, followed by hellbenders from the first‐generation Zoo breeding group, then hellbenders from the second‐generation Zoo breeding group (Figure 5‐5). River of origin was a significant predictor of the proportion of total mortality (*χ*² = 73.08, *p* < 0.001; Figure 4‐4) with Current River and North Fork of the White River individuals having significantly higher proportion of total mortality relative to Eleven Point River individuals. Egg origin was also a significant predictor of the proportion of total mortality (*χ*² = 188.47, *p* < 0.001; Figure 5‐3) with wild bred individuals having a lower proportion of total mortality relative to Zoo‐bred individuals. Further exploration of the breeding scenarios determined that wild‐bred hellbenders had the lowest larval mortality, followed by hellbenders from the first‐generation Zoo breeding group, then hellbenders from the second‐generation Zoo breeding group (Figure 5‐6).

## 4 Discussion

Our study examined biological outcomes from 9 years of Ozark hellbender conservation breeding and head‐starting efforts by the Zoo, MDC, and their partners. We observed significant differences in larval growth, long‐term growth, and proportional mortality for Zoo‐reared Ozark hellbenders based on river of origin and egg origin. When river of origin was a significant predictor of Ozark hellbender larval or long‐term growth, North Fork of the White River individuals tended to weigh more or measure longer than Eleven Point River or Current River individuals, analogous to body size observations from the wild and the genetic relatedness of these populations. Egg origin also influenced the long‐term growth of Ozark hellbenders, likely because of changing priorities for population augmentation releases as hellbenders reach sexual maturity. Mortality of Zoo‐reared Ozark hellbenders was often predicted by river of origin and egg origin. River of origin was a significant predictor of mortality across all life stages while egg origin was significant for egg, larva, and total mortality. Specifically, Zoo‐bred hellbenders had significantly higher proportional mortality relative to wild‐bred hellbenders. Ultimately, our results provide important baseline data for the propagation program efforts and build upon existing evidence supporting biologically distinct Ozark hellbender populations in Missouri.

The observed differences in Ozark hellbender larval and long‐term growth between the rivers of origin provide evidence of potential biological differences in body size and/or growth rate between Ozark hellbender populations. Our results indicate that Current River and Eleven Point River hellbenders at the Zoo tend to be smaller than North Fork of the White River hellbenders. The patterns we observed at the Zoo also appear consistent with records from the wild. Historic data is limited for the Current River; however, surveys from the late 1970s and early 1980s, indicate that North Fork of the White River individuals had a higher maximum (55.1 cm vs. 45.1 cm), minimum (17.2 cm vs. 11.9 cm), and average total length (approximately 35.0–39.9 vs. 30.0–34.9 cm), relative to Eleven Point River individuals (Peterson et al. 1983, 1988; Wheeler et al. 2003). Most professionals now agree that declines in Ozark hellbender populations likely began before these surveys or during these earlier surveys and that age structures shifted towards larger individuals with few or no juvenile hellbenders being detected (Wheeler et al. 2003; U.S. Fish and Wildlife Service 2020). Thus, it is possible that these observations were already depicting this skew, but comprehensive body length data for these rivers before these studies does not exist. The earliest records of Current River hellbender total body lengths are from 1992, which is well into the documented decline and could mean that the body lengths observed were already skewed towards larger individuals (Ziehmer and Johnson 1992; Wheeler et al. 2003; U.S. Fish and Wildlife Service 2020). Despite this, the pattern observed during that 1992 study also appears consistent with our results with maximum total lengths of 38.0 cm, minimum total lengths of 11.5 cm, and average total lengths of approximately 33.0–38.0 cm (Ziehmer and Johnson 1992). The persistence of natural variations in body size under the consistent environmental conditions of the Zoo indicates that local adaptation of Ozark hellbender populations towards particular body sizes and/or growth rates likely underlies the growth patterns rather than biological responses to different environmental conditions between the wild river populations. This is also supported by our finding that size differences between rivers of origin were observed across the whole size range (i.e., smallest, average, and largest) of larval individuals, which could indicate that the larger body sizes may be driven by underlying physiological differences in growth rates.

Previous genetic analyses would also suggest that our larval and long‐term growth results are indicative of local adaptation by river of origin. Overall, there is low genetic relatedness between Ozark hellbender populations, though Eleven Point River and Current River populations show higher levels of genetic relatedness as do North Fork of the White River and other White River tributary populations (Sabatino and Routman 2009; Crowhurst et al. 2011; Tonione, Johnson, and Routman 2011; Hime 2017). The results of these genetic analyses are consistent with our results of Current River and Eleven Point River individuals exhibiting more similar larval and long‐term growth responses relative to North Fork of the White River individuals. Our results, when combined with observed differences in body size (Peterson et al. 1983, 1988; Ziehmer and Johnson 1992; Wheeler et al. 2003), breeding seasons (Peterson et al. 1983, 1989; Briggler and Johnson 2021), and genetic relatedness (Routman 1993; Sabatino and Routman 2009; Crowhurst et al. 2011; Tonione, Johnson, and Routman 2011; Hime 2017) between Ozark hellbender populations, indicate that North Fork of the White River individuals are distinct from Eleven Point River and Current River individuals and that conservation efforts should continue to preserve these distinct lineages.

Egg origin was not a significant determinant for any larval growth responses but was for most long‐term growth responses with Zoo‐bred hellbenders tending to be smaller than wild‐bred hellbenders. This was unexpected because of the standardized care received by all nonbreeding Ozark hellbenders (Ettling et al. 2013). Yet, egg origin was a significant predictor for long‐term average weight and all of the total length growth responses. Further examination of these data showed consistent long‐term growth patterns for both egg origins until the hellbenders reached about 30 cm, where the divergence between egg origins becomes increasingly predominant (Figure 3). Female and male hellbenders are considered to be sexually mature at 37–39 and 30 cm, respectively (Bishop 1941; Dundee and Dundee 1965; Nickerson and Mays 1973; Taber, Wilkinson, and Topping 1975; Peterson et al. 1983). Thus, these results likely reflect nonrandom releases of sexually mature wild and Zoo‐bred hellbenders due to tradeoffs between maintaining genetic diversity at the Zoo and releasing optimal individuals into the wild. In practice, the Zoo often maintains large wild‐bred hellbenders for breeding purposes to maintain genetic diversity in the brood stock. Conversely, the MDC preferentially selects large Zoo‐bred hellbenders for release to improve survival odds following release while maintaining smaller Zoo‐bred individuals to release them at a more favorable size. This nonrandom approach to hellbender release post‐sexual maturity can explain the lack of significance for egg origin in larval growth parameters and the influence of egg origin to our long‐term growth responses.

In addition to differences in larval and long‐term growth by river of origin, we also observed differences in mortality by river of origin. Current River and North Fork of the White River hellbenders had higher proportional egg, hatchling, and total mortality than Eleven Point River hellbenders. For proportional larval mortality, Current River individuals had the highest rate of mortality, followed by Eleven Point River hellbenders, then North Fork of the White River hellbenders. Additionally, Current River hellbenders also had the highest rate of larval mortality amongst Zoo‐bred hellbenders. One potential explanation for this is unequally reduced survival due to population‐specific local adaptations. Several common garden and reciprocal transplant experiments have found survival and fitness benefits for locally adapted amphibian populations (Gomez‐Mestre et al. 2003; Olsson and Uller 2003; Brady 2012; Bachmann and Van Buskirk 2021). It is not possible to replicate the conditions of a natural river perfectly; therefore, it is possible that the environmental conditions of the Zoo are more or less favorable for certain rivers of origin based on the local adaptations of the population. In other words, if the conditions at the Zoo are more closely aligned to the conditions of a particular river of origin, perhaps Ozark hellbenders from those rivers experience improved survival rates. For example, differences in water quality parameters such as temperature variability, discharge, dissolved oxygen, and/or ionic concentrations could influence Ozark hellbender survival, and the conditions at the Zoo for these parameters may be more optimal for certain populations. However, it is not currently clear how these parameters affect Ozark hellbender survival, if at all, across different life stages. Moreover, many of these parameters fluctuate daily and seasonally, and detailed differences between water quality at the Zoo and the natural river systems are less clear at these temporal scales.

Another possible hypothesis is that differing levels of genetic diversity between Ozark hellbender rivers of origin resulted in higher or lower rates of mortality. Several genetic studies have indicated low levels of genetic diversity within hellbender populations (Sabatino and Routman 2009, Crowhurst et al. 2011, Tonione, Johnson, and Routman 2011; Hime 2017). A lack of genetic diversity, combined with declining populations and reduced recruitment, leaves hellbender populations vulnerable to Allee effects, genetic drift, and inbreeding depression (Allee and Bowen 1932; Boyce 1992; Lynch, Conery, and Burger 1995; Keller and Waller 2002; Reed and Frankham 2003). All of which can further reduce growth, survival, reproduction, and population‐level susceptibility to environmental catastrophes, disease, and extirpation (Allee and Bowen 1932; Boyce 1992; Lynch, Conery, and Burger 1995; Keller and Waller 2002; Reed and Frankham 2003; Allentoft and O'Brien 2010). For amphibians, reduced genetic variability can strongly influence the fitness of early life stages (Halverson, Skelly, and Caccone 2006; Richter and Nunziata 2014). While we are unable to compare the genetic diversity of clutches without genetic testing, it is possible that the differential survival by river of origin we observed could be the result of underlying differences in genetic diversity in breeding groups and/or the rivers of origin. Interestingly, there is evidence that the reduced survival and fitness caused by genetic inbreeding is often higher in natural populations than captive populations due to additional sources of mortality for less fit individuals (Crnokrak and Roff 1999; Halverson, Skelly, and Caccone 2006). Thus, the continued Ozark hellbender monitoring efforts in Missouri rivers by the MDC could help confirm whether mortality rates differ between hellbender populations in concordance with known levels of genetic diversity.

We found a significant influence of egg origin for Zoo‐reared Ozark hellbender egg, larva, and total mortality. In all of these cases, Zoo‐bred Ozark hellbenders had significantly higher proportional mortality than wild‐bred Ozark hellbenders. Firstly, we acknowledge that the proportional egg mortality response could be confounded by egg origin. The number of eggs collected in the Zoo and the wild are likely to be different based on the amount of time between oviposition and the eggs being collected and counted. Zoo‐laid egg counts are likely to provide a more accurate estimate of oviposited egg counts due to early detection and counting as well as reduced accumulated losses from infertility, cannibalism, predation, and congenital issues that are incompatible with life. Though not explicitly documented, visual inspection of egg development stages of Zoo‐bred and wild‐bred eggs suggests that Zoo‐bred eggs are typically less developed than wild‐bred eggs at the time of collection, which is likely due to spending less time in the artificial streams—at least 12–14 days following oviposition and fertilization—than wild‐bred eggs spend in native rivers before collection. We estimated that wild‐bred eggs brought to the Zoo completed approximately half of their development, on average, before collection because Zoo‐bred eggs hatch in an average of 51 days while wild‐bred origin eggs hatch in an average of 26 days after entering the Zoo. This means that wild‐bred eggs have approximately double the time for unaccounted egg losses as Zoo‐bred eggs and are entering the Zoo at a much more advanced developmental stage, which increases egg survival and hatching success (Civiello et al. 2018). Moreover, wild‐bred eggs experience parental care from the brooding male for a longer duration during development than Zoo‐bred eggs, which can improve developmental outcomes and hatching success (Okada, Fukuda, and Takahashi 2015; Takahashi, Okada, and Fukuda 2017; Settle, Briggler, and Mathis 2018; Terry et al. 2019). Thus, as expected, wild‐bred eggs had significantly lower proportional egg mortality relative to Zoo‐bred eggs.

While we expected the proportional egg mortality response to be heavily influenced by egg origin, we did not anticipate differences persisting into later life stages. This led us to investigate the relationship further and examine differences between the different breeding group generations at the Zoo. We confirmed that proportional mortality was highest for hellbenders from the second‐generation breeding group, followed by hellbenders from the first‐generation breeding group, and hellbenders from the wild have the lowest proportional mortality rates. Given that nonbreeding Ozark hellbenders experience the same conditions at the Zoo after wild‐bred eggs are brought to the Zoo, it is unclear what mechanism might be responsible for later life stage deaths. One hypothesis is that there might be long‐term benefits to the parental care that males provide during egg development. Male hellbenders exhibit several behaviors that could benefit egg success in the long‐term. The first is tail fanning of the eggs (Okada, Fukuda, and Takahashi 2015; Settle, Briggler, and Mathis 2018). This behavior likely helps to oxygenate the eggs, which is crucial for proper development (Okada, Fukuda, and Takahashi 2015; Settle, Briggler, and Mathis 2018). Another important parental care behavior is agitation of the nest area, which is suspected to reduce the incidence of developmental abnormalities and remove debris from the nest (Okada, Fukuda, and Takahashi 2015; Takahashi, Okada, and Fukuda 2017; Settle, Briggler, and Mathis 2018; Terry et al. 2019). The Zoo replicates fanning and agitation of the eggs with pumps to create water flow in the enclosures and by manually “rocking” the eggs 3‐4 times daily (Pedigo et al. 2021). The Zoo has found this to be necessary for successful embryo development and hatching success. However, it is possible that the quality or frequency of movement and oxygenation is not equivalent to that of the brooding male such that Zoo‐bred eggs experience lasting effects. A third potentially beneficial behavior is the brooding male's propensity for hygienic filial cannibalism of the eggs, which is when brooding males selectively consume eggs that are unfertilized, dead, or infected (Okada, Fukuda, and Takahashi 2015). Filial cannibalism has been noted several times in both *Cryptobranchus* and the closely related *Andrias* genus (Smith 1907, 1912; Okada, Fukuda, and Takahashi 2015; Takahashi, Okada, and Fukuda 2017; Settle, Briggler, and Mathis 2018; Unger and Williams 2018; Terry et al. 2019). However, the observation of hygienic filial cannibalism provides a potential mechanism by which eggs that will fail to thrive can be selectively removed and not be accounted for in the egg or later life stage proportional mortality responses. It also could help explain potential differences between Zoo‐ and wild‐bred Ozark hellbenders given the disparity in time spent with brooding males. For example, hygienic filial cannibalism is reported to reduce the spread of *Saprolegnia*, also known as water mold, within Cryptobranchid egg clutches (Okada, Fukuda, and Takahashi 2015; Takahashi, Okada, and Fukuda 2017). *Saprolegnia* infection can lead to mortality or accelerated development of amphibian eggs, with long‐term effects in later life stages (Warkentin, Currie, and Rehner 2001; Gomez‐Mestre, Touchon, and Warkentin 2006; Uller, Sagvik, and Olsson 2009; Fernández‐Benéitez et al. 2011). Furthermore, *Saprolegnia* infections have been linked to mortality of Cryptobranchid eggs in captivity and the wild (Smith 1907, 1912; Bodinof 2010; Unger and Mathis 2013; Okada, Fukuda, and Takahashi 2015; Takahashi, Okada, and Fukuda 2017). Thus, if brooding males have the ability to identify and selectively consume dead, sub‐lethally infected eggs, and/or vulnerable surrounding eggs, perhaps egg mortality and/or adverse developmental consequences can be minimized. To replicate hygienic filial cannibalism, the Zoo staff manually remove eggs exhibiting signs of infertility, infections such as *Saprolegnia*, and eggs with severe congenital defects as a preventative measure. However, it is possible that invisible chemical indicators or sub‐lethal infections, for example, are missed. It is also interesting to note that brooding *Andrias japonicas* males continue to perform fanning, agitation, and selective hygienic filial cannibalism behaviors post‐hatching, and this attendance improves hatchling success (Takahashi, Okada, and Fukuda 2017). While no Zoo‐reared hellbenders hatch with their respective brooding male, this research highlights how extended parental care might increase reproductive success beyond the egg life stage. In all, the extended time that wild‐bred eggs spend with brooding males could promote successful egg development and reduce adverse developmental outcomes such as mortality, developmental abnormalities, and accelerated development, which might otherwise result in eventual or inevitable deaths in later life stages. Future research could leave eggs with brooding males for variable lengths of time to assess the importance of parental care to long‐term mortality.

An alternative hypothesis is that parental allocation of resources to reproduction are different between breeding group generations as a result of maturity. Female size is biologically related to resource allocation for reproduction with larger females being able to produce larger clutches, larger ovums, and eggs that develop more slowly and hatch at a more advanced developmental stage (Salthe 1969; Topping and Ingersol 1981; Duellman and Trueb 1986; Nussbaum 1987). First‐generation breeding group females at the Zoo were older and had higher average total lengths (47.0 cm) than second‐generation breeding females (42.8 cm), which resulted in first‐generation breeding group females producing more eggs each reproductive year, on average, that develop for longer, on average, than eggs produced by less mature second‐generation females (Macklem et al. 2024). Similarly, the more mature first‐generation males might have been better able to fertilize, brood, and defend the eggs from predation as well as perform energetically costly behaviors such as egg fanning and agitation than less mature second‐generation males. While we didn't quantify differences in male parental care during the time when eggs were still with brooding males at the Zoo, we found evidence to suggest that first generation males were more successful at fertilizing eggs, reducing egg infertility and, therefore, proportional egg mortality (Macklem et al. 2024). In amphibians, increased resource allocation in the egg life stage often corresponds to amplifying benefits through later life stages. For example, more developed hatchlings often experience reduced predation threats, increased availability of food resources, and further acceleration of growth (Kaplan and Kaplan 1980; Sih and Moore 1993; Moore, Newton, and Sih 1996; Pagnucco, Paszkowski, and Scrimgeour 2011). Thus, it is possible that the benefits experienced by hellbenders born from the more mature first‐generation breeding group extend to later life stages and result in reduced proportional egg, hatchling, larval, and total mortalities relative to hellbenders from the second‐generation breeding group. It is also possible that these body size and reproductive resource allocation differences extend to wild‐bred clutches since the age structures of wild populations have shifted towards larger, older individuals as populations have declined (U.S. Fish and Wildlife Service 2020; Wheeler et al. 2003). Thus, if wild‐bred eggs came from larger, more mature hellbenders than the Zoo's breeding groups, perhaps these hellbenders have lower proportional egg, larva, and total mortality due to advantages from increased resource allocation as eggs.

Conservation breeding and head‐starting initiatives allow for controlled breeding and maintenance of genetic diversity and distinctness (Ralls, Ballou, and Templeton 1988; Crnokrak and Roff 1999; Browne et al. 2011, Gratwicke and Murphy 2017) while augmenting wild populations with individuals that are less vulnerable to mortality and/or able to contribute to recruitment (Anderson, Hassinger, and Dalrymple 1971; Griffiths and Pavajeau 2008; Browne et al. 2011; Harding, Griffiths, and Pavajeau 2016; Thomas et al. 2018). The forethought of agency staff to understand the genetic structure of Ozark hellbender populations led to the maintenance of genetically distinct populations of this endangered subspecies at the Zoo, and the data from these efforts have provided important baseline information for *ex situ* care as well as corroborative data to contribute to targeted *in situ* conservation and management efforts for Ozark hellbenders.

## Ethics Statement

Research was conducted in compliance with applicable animal care guidelines and appropriate permits. Hellbender eggs from the wild were collected by J. Briggler of the Missouri Department of Conservation under the authority of the Wildlife Code of Missouri and federal permit.

## Conflicts of Interest

The authors declare no conflicts of interest.

## Data Availability

The data are not publicly available due to conservation concerns for the species.

## References

[zoo21870-bib-0001] Allee, W. C. , and E. S. Bowen . 1932. “Studies in Animal Aggregations: Mass Protection Against Colloidal Silver Among Goldfishes.” Journal of Experimental Zoology 61, no. 2: 185–207.

[zoo21870-bib-0002] Allentoft, M. E. , and J. O'Brien . 2010. “Global Amphibian Declines, Loss of Genetic Diversity, and Fitness: A Review.” Diversity 2: 47–71. 10.3390/d2010047.

[zoo21870-bib-0003] Anderson, J. D. , D. D. Hassinger , and G. H. Dalrymple . 1971. “Natural Mortality of Eggs and Larvae of *Ambystoma t. tigrinum* .” Ecology 52, no. 6: 1107–1112.

[zoo21870-bib-0004] Bachmann, J. C. , and J. Van Buskirk . 2021. “Adaptation to Elevation But Limited Local Adaptation in an Amphibian.” Evolution 75, no. 4: 956–969. 33063864 10.1111/evo.14109

[zoo21870-bib-0005] Bishop, S. C. 1941. “The Salamanders of New York.” New York State Museum Bulletin 324: 1–365.

[zoo21870-bib-0006] Bodinof, C. M. 2010. Translocation and Conservation of Hellbenders (*Cryptobranchus alleganiensis*) in Missouri. Columbia, Missouri, USA: University of Missouri.

[zoo21870-bib-0007] Boyce, M. S. 1992. “Population Viability Analysis.” Annual Review of Ecology and Systematics 23: 481–497.

[zoo21870-bib-0008] Brady, S. P. 2012. “Road to Evolution? Local Adaptation to Road Adjacency in an Amphibian (*Ambystoma maculatum*).” Scientific Reports 2: 235. 10.1038/srep00235. 22355748 PMC3267261

[zoo21870-bib-0009] Briggler, J. T. , T. Crabill , K. J. Irwin , et al. 2012. Propagation, Augmentation, and Reintroduction Plan for the Ozark Hellbender (*Cryptobranchus alleganiensis bishopi*). Jefferson City, Missouri, USA: Ozark Hellbender Propagation Committee.

[zoo21870-bib-0010] Briggler, J. T. , T. Crabill , K. J. Irwin , C. Davidson , J. Utrup , and A. Salveter . 2010. Hellbender Conservation Strategy: An Action Plan for the Recovery of the Ozark and Eastern Hellbender in the Ozark Highlands of Missouri and Arkansas. Jefferson City, Missouri, USA: Ozark Hellbender Working Group.

[zoo21870-bib-0011] Briggler, J. T. , and T. R. Johnson . 2021. “Family Cryptobranchidae, Hellbenders and Giant salamanders.” In The Amphibians and Reptiles of Missouri, edited by L. Archer , (revised and expanded 3rd ed.), 48–50. Jefferson City, Missouri, USA: Missouri Department of Conservation.

[zoo21870-bib-0012] Browne, R. K. , K. Wolfram , G. García , M. F. Bagaturov , and J. J. M. Pereboom . 2011. “Zoo‐Based Amphibian Research and Conservation Breeding Programs.” Amphibian and Reptile Conservation 5, no. 3: 1–14 (e28).

[zoo21870-bib-0013] Burnham, K. P. , and D. R. Anderson . 2002. Model Selection and Multimodel Inference: A Practical Information‐Theoretic Approach (2nd ed.). New York, USA: Springer.

[zoo21870-bib-0014] Civiello, J. A. , T. J. Bruce , S. J. Brisco , and J. T. Briggler . 2018. “Propagation of Eastern Hellbenders Cryptobranchus alleganiensis alleganiensisin a Recirculating Aquaculture System at Shepherd of the Hills State Fish Hatchery.” North American Journal of Aquaculture 81, no. 4: 281–290. 10.1002/naaq.10065.

[zoo21870-bib-0015] Crnokrak, P. , and D. A. Roff . 1999. “Inbreeding Depression in the Wild.” Heredity 83: 260–270. 10504423 10.1038/sj.hdy.6885530

[zoo21870-bib-0016] Crowhurst, R. S. , K. M. Faries , J. Collantes , J. T. Briggler , J. B. Koppelman , and L. S. Eggert . 2011. “Genetic Relationships of Hellbenders in the Ozark Highlands of Missouri and Conservation Implications for the Ozark Subspecies (*Cryptobranchus alleganiensis bishopi*).” Conservation Genetics 12, no. 3: 637–646. 10.1007/s10592-010-0170-0.

[zoo21870-bib-0017] Duellman, W. E. , and L. Trueb . 1986. Biology of Amphibians. New York, USA: McGraw‐Hill.

[zoo21870-bib-0018] Dundee, H. A. , and D. S. Dundee . 1965. “Observations on the Systematics and Ecology of *Cryptobranchus* From the Ozark Plateaus of Missouri and Arkansas.” Copeia 1965, no. 3: 369–370. 10.2307/1440805.

[zoo21870-bib-0019] Ettling, J. , M. D. Wanner , C. D. Schuette , S. L. Armstrong , A. S. Pedigo , and J. T. Briggler . 2013. “Captive Reproduction and Husbandry of Adult Ozark Hellbenders, *Cryptobranchus alleganiensis bishopi* .” Herpetological Review 44, no. 4: 605–610.

[zoo21870-bib-0020] Fernández‐Benéitez, M. J. , M. E. Ortiz‐Santaliestra , M. Lizana , and J. Diéguez‐Uribeondo . 2011. “Differences in Susceptibility to Saprolegnia Infections Among Embryonic Stages of Two Anuran Species.” Oecologia 165, no. 3: 819–826. 21197546 10.1007/s00442-010-1889-5

[zoo21870-bib-0021] Gomez‐Mestre, I. , M. Tejedo , I. Gomez‐Mestre , and M. Tejedo . 2003. “Local Adaptation of an Anuran Amphibian to Osmotically Stressful Environments.” Evolution 57, no. 8: 1889–1899. 14503630 10.1111/j.0014-3820.2003.tb00596.x

[zoo21870-bib-0022] Gomez‐Mestre, I. , J. C. Touchon , and K. M. Warkentin . 2006. “Amphibian Embryo and Parental Defenses and a Larval Predator Reduce Egg Mortality From Water Mold.” Ecology 87, no. 10: 2570–2581. 17089665 10.1890/0012-9658(2006)87[2570:aeapda]2.0.co;2

[zoo21870-bib-0023] Gratwicke, B. , and J. B. Murphy . 2017. “History of Captive Management and Conservation Amphibian Programs Mostly in Zoos and Aquariums. Part II‐Salamanders and Caecilians.” Herpetological Review 48, no. 2: 474–486.

[zoo21870-bib-0024] Green, N. B. , and T. K. Pauley . 1987. Amphibians and Reptiles in West Virginia. Pittsburgh, Pennsylvania, USA: University of Pittsburgh Press.

[zoo21870-bib-0025] Griffiths, R. A. , and L. Pavajeau . 2008. “Captive Breeding, Reintroduction, and the Conservation of Amphibians.” Conservation Biology 22, no. 4: 852–861. 18616746 10.1111/j.1523-1739.2008.00967.x

[zoo21870-bib-0026] Halverson, M. , D. Skelly , and A. Caccone . 2006. “Inbreeding Linked to Amphibian Survival in the Wild But Not in the Laboratory.” Journal of Heredity 97, no. 5: 499–507. 16957048 10.1093/jhered/esl019

[zoo21870-bib-0027] Harding, G. , R. A. Griffiths , and L. Pavajeau . 2016. “Developments in Amphibian Captive Breeding and Reintroduction Programs.” Conservation Biology 30, no. 2: 340–349. 10.1111/cobi.12612. 26306460

[zoo21870-bib-0028] Hime, P. M. 2017. Genomic Perspectives on Aamphibian Evolution Across Multiple Phylogenetic Scales. Lexington, Kentucky, USA: University of Kentucky. 10.13023/ETD.2017.284.

[zoo21870-bib-0029] Junge, R. E. 2011. “Hellbender Medicine.” In Fowler*'s Zoo and Wild Animal Medicine: Current Therapy* , edited by R. E. Miller and M. Fowler , 260–264. Philadelphia, Pennsylvania, USA: Saunders.

[zoo21870-bib-0030] Kaplan, R. H. , and R. H. Kaplan . 1980. “The Implications of Ovum Size Variability for Offspring Fitness and Clutch Size Within Several Populations of Salamanders (*Ambystoma*).” Evolution 34: 51–64. 28563211 10.1111/j.1558-5646.1980.tb04788.x

[zoo21870-bib-0031] Keller, L. F. , and D. M. Waller . 2002. “Inbreeding Effects in Wild Populations.” TRENDS in Ecology & Evolution 17, no. 5: 230–241.

[zoo21870-bib-0032] Kucuktas, H. , B. K. Wagner , R. Shopen , M. Gibson , R. A. Dunham , and Z. Liu . 2001. “Genetic Analysis of Ozark Hellbenders Utilizing RAPD Markers.” Proceedings of the Annual Conference of the Southeastern Association of Fish and Wildlife Agencies 55: 126–137.

[zoo21870-bib-0033] Luo, Q. , F. Tong , Y. Song , H. Wang , M. Du , and H. Ji . 2018. “Observation of the Breeding Behavior of the Chinese Giant Salamander (*Andrias davidianus*) Using a Digital Monitoring System.” Animals 8, no. 161: 161. 30257506 10.3390/ani8100161 PMC6211081

[zoo21870-bib-0034] Lynch, M. , J. Conery , and R. Burger . 1995. “Mutation Accumulation and the Extinction of Small Populations.” American Naturalist 146, no. 4: 489–518.

[zoo21870-bib-0080] Macklem, D. C. , L. Augustine , M. D. Wanner , et al. 2024. “Egg Production, Egg Development, and Mortality of Zoo‐Bred Ozark Hellbenders (Cryptobranchus alleganiensis bishopi).” *Zoo Biology*. 10.1002/zoo.21869. PMC11802487 39588560

[zoo21870-bib-0035] Moore, R. D. , B. Newton , and A. Sih . 1996. “Delayed Hatching as a Response of Streamside Salamander Eggs to Chemical Cues From Predatory Sunfish.” Oikos 77: 331–335.

[zoo21870-bib-0036] Nickerson, M. A. , and C. E. Mays . 1973. “The Hellbenders: North American ‘Giant Salamanders’.” Milwaukee Public Museum Publications in Biology and Geology 1: 1–106.

[zoo21870-bib-0037] Nussbaum, R. A. 1987. “Parental Care and Egg Size in Salamanders: An Examination of the Safe Harbor Hypothesis.” Population Ecology 29, no. 1: 27–44.

[zoo21870-bib-0038] Okada, S. , Y. Fukuda , and M. K. Takahashi . 2015. “Paternal Care Behaviors of Japanese Giant Salamander *Andrias japonicus* in Natural Populations.” Journal of Ethology 33: 1–7. 10.1007/s10164-014-0413-5.

[zoo21870-bib-0039] Olsson, M. , and T. Uller . 2003. “Thermal Environment, Survival and Local Adaptation in the Common Frog, *Rana temporaria* .” Evolutionary Ecology Research 5: 431–437.

[zoo21870-bib-0040] Pagnucco, K. S. , C. A. Paszkowski , and G. J. Scrimgeour . 2011. “Wolf in Sheep's Clothing: Effects of Predation by Small‐Bodied Fish on Survival and Behaviour of Salamander Larvae.” Ecoscience 18, no. 1: 70–78.

[zoo21870-bib-0041] Pedigo, A. S. , K. R. Noble , P. L. Ihrig‐Bueckendorf , et al. 2021. Saint Louis Zoo Hellbender Husbandry Manual Saint Louis Zoo WildCare Institute Ron Goellner Center for Hellbender Conservation. Missouri, USA: Saint Louis Zoo.

[zoo21870-bib-0042] Peterson, C. L. , C. A. Ingersol , and R. F. Wilkinson . 1989. “Winter Breeding of *Cryptobranchus alleganiensis bishopi* in Arkansas.” Copeia 1989, no. 4: 1031–1035.

[zoo21870-bib-0043] Peterson, C. L. , D. E. Metter , B. T. Miller , R. F. Wilkinson , and M. S. Topping . 1988. “Demography of the Hellbender *Cryptobranchus alleganiensis* in the Ozarks.” American Midland Naturalist 119, no. 2: 291–303.

[zoo21870-bib-0044] Peterson, C. L. , R. F. Wilkinson, Jr. , M. S. Topping , and D. E. Metter . 1983. “Age and Growth of the Ozark Hellbender (*Cryptobranchus alleganiensis bishopi*).” Copeia 1983, no. 1: 225–231.

[zoo21870-bib-0045] Petranka, J. W. 1998. “Family Cryptobranchidae: Hellbender and Giant Salamanders.” In Salamanders of the United States and Canada, edited by P. Strupp , 139–144. Washington, D. C., USA: Smithsonian Institution Press.

[zoo21870-bib-0046] Ralls, K. , J. D. Ballou , and A. Templeton . 1988. “Estimates of Lethal Equivalents and the Cost of Inbreeding in Mammals.” Conservation Biology 2, no. 2: 185–193.

[zoo21870-bib-0047] Reed, D. H. , and R. Frankham . 2003. “Correlation Between Fitness and Genetic Diversity.” Conservation Biology 17, no. 1: 230–237.

[zoo21870-bib-0048] Richter, S. C. , and S. O. Nunziata . 2014. “Survival to Metamorphosis Is Positively Related to Genetic Variability in a Critically Endangered Amphibian Species.” Animal Conservation 17: 265–274.

[zoo21870-bib-0049] Routman, E. 1993. “Mitochondrial DNA Variation in *Cryptobranchus alleganiensis*, a Salamander With Extremely Low Allozyme Diversity.” Copeia 1993, no. 2: 407–416.

[zoo21870-bib-0051] Sabatino, S. J. , and E. J. Routman . 2009. “Phylogeography and Conservation Genetics of the Hellbender Salamander (*Cryptobranchus alleganiensis*).” Conservation Genetics 10, no. 5: 1235–1246. 10.1007/s10592-008-9655-5.

[zoo21870-bib-0050] Saint Louis Zoo WildCare Institute . 2018. 2018 *Annual Report* . St. Louis, Missouri, USA: Saint Louis Zoo.

[zoo21870-bib-0052] Salthe, S. N. 1969. “Reproductive Modes and the Number and Sizes of Ova in the Urodeles.” American Midland Naturalist 81: 467–490.

[zoo21870-bib-0053] Settle, R. A. , J. T. Briggler , and A. Mathis . 2018. “A Quantitative Field Study of Paternal Care in Ozark Hellbenders, North America's Giant Salamanders.” Journal of Ethology 36, no. 3: 235–242. 10.1007/s10164-018-0553-0.

[zoo21870-bib-0054] Sih, A. , and R. D. Moore . 1993. “Delayed Hatching of Salamander Eggs in Response to Enhanced Larval Predation Risk.” American Naturalist 142: 947–960. 10.1086/285583 19425943

[zoo21870-bib-0055] Smith, B. G. 1907. “The Life History and Habits of *Cryptobranchus allegheniensis* .” The Biological Bulletin 13, no. 1: 5–39.

[zoo21870-bib-0056] Smith, B. G. 1912. “The Embryology of *Cryptobranchus allegheniensis*, Including Comparisons With Some Other Vertebrates I. Introduction; the History of the Egg Before Cleavage.” Journal of Morphology 23, no. 1: 61–157.

[zoo21870-bib-0057] Taber, C. A. , R. F. Wilkinson, Jr. , and M. S. Topping . 1975. “Age and Growth of Hellbenders in the Niangua River, Missouri.” Copeia 1975, no. 4: 633–639.

[zoo21870-bib-0058] Takahashi, M. K. , S. Okada , and Y. Fukuda . 2017. “From Embryos to Larvae: Seven‐Month‐Long Paternal Care by Male Japanese Giant Salamander.” Journal of Zoology 302, no. 1: 24–31. 10.1111/jzo.12433.

[zoo21870-bib-0059] Templeton, A. R. , K. Shaw , E. Routman , and S. K. Davis . 1990. “The Genetic Consequences of Habitat Fragmentation.” Annals of the Missouri Botanical Garden 77, no. 1: 13–27.

[zoo21870-bib-0060] Terry, J. , Y. Taguchi , J. Dixon , K. Kuwabara , and M. K. Takahashi . 2019. “Preoviposition Paternal Care in a Fully Aquatic Giant Salamander: Nest Cleaning by a Den Master.” Journal of Zoology 307, no. 1: 36–42. 10.1111/jzo.12615.

[zoo21870-bib-0061] Thomas, P. , D. M. Boyer , D. A. Oehler , S. Silver , and L. Perrotti . 2018. “Headstarting as a Conservation Strategy for Threatened and Endangered Species.” In Scientific Foundations of Zoos and Aquariums: Their Role in Conservation and Research, edited by A. B. Kaufman , M. J. Bashaw , and T. L. Maple , 91–111. Cambridge, UK: Cambridge University Press.

[zoo21870-bib-0062] Tonione, M. , J. R. Johnson , and E. J. Routman . 2011. “Microsatellite Analysis Supports Mitochondrial Phylogeography of the Hellbender (*Cryptobranchus alleganiensis*).” Genetica 139, no. 2: 209–219. 10.1007/s10709-010-9538-9. 21161568

[zoo21870-bib-0063] Topping, M. S. , and C. A. Ingersol . 1981. “Fecundity in the Hellbender, *Cryptobranchus alleganiensis* .” Copeia 1981, no. 4: 873–876.

[zoo21870-bib-0064] Uller, T. , J. Sagvik , and M. Olsson . 2009. “Pre‐Hatching Exposure to Water Mold Reduces Size at Metamorphosis in the Moor Frog.” Oecologia 160: 9–14. 19189128 10.1007/s00442-009-1280-6

[zoo21870-bib-0065] Unger, S. , C. M. Bodinof Jachowski , L. Diaz , and L. A. Williams . 2020. “Shelter Guarding Behavior of the Eastern Hellbender (*Cryptobranchus alleganiensis alleganiensis*) in North Carolina Streams.” Southeastern Naturalist 19, no. 4: 742–758.

[zoo21870-bib-0066] Unger, S. , Z. C. Hull , L. Diaz , J. D. Groves , L. A. Williams , and C. M. Bodinof Jachowski . 2021. “Underwater Video Cameras Allow for Detection of North American Giant Salamanders (*Cryptobranchus alleganiensis alleganiensis*) in Both Captive and Wild Streams.” Aquaculture and Fisheries 6, no. 1: 106–110.

[zoo21870-bib-0067] Unger, S. D. , and A. Mathis . 2013. “Larval Growth and the Potential for Head‐Starting of Eastern and Ozark Hellbenders (*Cryptobranchus alleganiensis alleganiensis* and *C. a. bishopi*).” Herpetological Review 44, no. 1: 89–91.

[zoo21870-bib-0068] Unger, S. D. , and R. N. Williams . 2018. “Genetic Confirmation of Filial Cannibalism in North America's Giant Salamander, the Eastern Hellbender *Cryptobranchus alleganiensis alleganiensis* .” Ethology Ecology & Evolution 30, no. 2: 187–193. 10.1080/03949370.2017.1342696.

[zoo21870-bib-0069] U.S. Fish and Wildlife Service . 2011. “Endangered and Threatened Wildlife and Plants; Endangered Status for the Ozark Hellbender Salamander.” Federal Register 76, no. 194: 61956–61978.

[zoo21870-bib-0070] U.S. Fish and Wildlife Service . 2020. Biological Report for the Ozark Hellbender. Columbia, Missouri, USA: U.S. Fish and Wildlife Service.

[zoo21870-bib-0071] U.S. Fish and Wildlife Service . 2021. *Recovery Plan for the Ozark hellbender (Cryptobranchus alleganiensis bishopi)* . Bloomington, Minnesota, USA: U.S. Fish and Wildlife Service, Midwest Region.

[zoo21870-bib-0072] de Villemereuil, P. , O. E. Gaggiotti , M. Mouterde , and I. Till‐Bottraud . 2016. “Common Garden Experiments in the Genomic Era: New Perspectives and Opportunities.” Heredity 116, no. 3: 249–254. 26486610 10.1038/hdy.2015.93 PMC4806574

[zoo21870-bib-0073] Warkentin, K. M. , C. R. Currie , and S. A. Rehner . 2001. “Egg‐Killing Fungus Induces Early Hatching of Red‐Eyed Treefrog Eggs.” Ecology 82, no. 10: 2860–2869.

[zoo21870-bib-0074] Wheeler, B. A. , E. Prosen , A. Mathis , and R. F. Wilkinson . 2003. “Population Declines of a Long‐Lived Salamander: A 20+‐Year Study of Hellbenders, *Cryptobranchus alleganiensis* .” Biological Conservation 109, no. 1: 151–156. 10.1016/S0006-3207(02)00136-2.

[zoo21870-bib-0075] Ziehmer, B. , and T. Johnson (1992). Status of the Ozark hellbender in Missouri. Jefferson City, Missouri, USA: Missouri Department of Conservation.

